# Dynamics of infection, vaccination and excess mortality during the COVID-19 pandemic among older individuals—a nationwide analysis

**DOI:** 10.1007/s10654-026-01414-1

**Published:** 2026-06-08

**Authors:** Eva A. S. Koster, Marije H. Sluiskes, Hein Putter, Frits R. Rosendaal, Astrid van Hylckama Vlieg, Mark G. J. de Boer, Liesbeth C. de Wreede

**Affiliations:** 1 Department of Biomedical Data Sciences, Leiden University Medical Center, Leiden, the Netherlands; 2 Department of Clinical Epidemiology, Leiden University Medical Center, Leiden, the Netherlands; 3 Leiden University Center for Infectious Diseases, Leiden University Medical Center, Leiden, the Netherlands

**Keywords:** COVID-19 pandemic, Excess mortality, Vaccination, Relative survival, Multi-state modelling

## Abstract

The Coronavirus Diseases (COVID-19) pandemic led to excess mortality in many countries, i.e., to more deaths than expected. Older individuals generally had higher absolute risks of excess death. The complex dynamics of infection, vaccination and excess mortality have not yet been captured in one comprehensive model. With nationwide and unselected data from Statistics Netherlands, we analyzed the impact of documented infection and vaccination on excess mortality during 2020 and 2021 in the Dutch population aged over 63 on 1 January 2020 (*n* = 3,826,770) by incorporating relative survival into a multi-state model considering COVID-19 (re)infection, vaccination and death. Background mortality was based on the observed mortality in 2015–2019 per sex, age and month of the year. All analyses were performed for the total cohort as well as stratified per sex and age category. The absolute excess mortality in 2020–2021 was 0.34% (95% confidence interval 0.32–0.37), comprising 4.41% of the observed mortality. It was higher in men (except in the youngest age group) and older individuals. Excess mortality occurred mostly during the first four weeks after a positive COVID-19 test, but also thereafter. If infection occurred after vaccination, excess mortality was still observed, but considerably less than without prior vaccination. These patterns were observed in all groups. In conclusion, these analyses demonstrate the substantial impact of COVID-19 on excess mortality during and after acute SARS-CoV-2 infection in individuals aged over 63 years. Moreover, the results show a reduction of excess mortality after vaccination for all groups in this cohort.

**Supplementary Information:**

The online version contains supplementary material available at https://doi.org/10.1007/s10654-026-01414-1.

## Introduction

During the Coronavirus Diseases (COVID-19) pandemic there was an increase in mortality in many countries, referred to as excess mortality [1, 2]. While the presence of excess mortality is well-documented, an accurate estimation of its magnitude is challenging, mainly because it is not expressed directly by the number of registered COVID-19 deaths. Correct estimation of the pandemic-related excess mortality is essential for informing the public, identifying high-risk groups and evaluating the appropriateness of the anti-COVID-19 measures and distribution of limited resources. All these elements are required to improve preparedness for future pandemics.

The absolute risk of COVID-19-related excess death was generally higher among persons aged 65 years or older [1]. This is primarily attributed to the relatively high case fatality rate of primary COVID-19 infections in this population [3]. After the introduction of mass vaccination, several studies showed less protection by SARS-CoV-2 vaccination against death from COVID-19 with advancing age [4, 5]. However, none of these studies reported the full sequence of events of vaccination, infection and excess mortality.

Therefore, the current study aimed to capture the complex dynamics of documented COVID-19 infection, vaccination and all-cause excess mortality in one comprehensive model by incorporating relative survival into an extensive multi-state model considering (re)infection, vaccination and death using the recently developed methodology by Manevski et al. [6]. We focused on the first two years of the pandemic in the Netherlands, considering all individuals who were or could have become 65 years or older during this period. For this, we used nationwide comprehensive data from Statistics Netherlands, which contains all registered positive COVID-19 test results and almost complete SARS-CoV-2 vaccination data in the Dutch population [7].

## Methods

To investigate excess mortality in the study period 2020–2021, the observed all-cause mortality during this period needs to be split into background and excess mortality, where the background mortality is the mortality as expected on the basis of historical data. Balancing between the variability in mortality between years and the trend of improving health care and life expectancy over the years, we chose the period 2015–2019 as the reference period.

### Data and study population

This nationwide cohort study used anonymized, individual-level data of 2015–2019 (reference period) and 2020–2021 (study period) for the Dutch population, provided by Statistics Netherlands. For the study period we included all registered persons living in the Netherlands at 1 January in 2020 or 2021 who were born before 1957. Because we did not have access to immigration and emigration dates, persons who immigrated to the Netherlands during 2020 were included in the dataset from 1 January 2021 onwards, while persons who emigrated during 2020 were censored on 1 January 2021, and migration during 2021 was not considered (Supplemental Fig. 1). All other surviving persons were administratively censored on 31 December 2021. For each year of the reference period 2015–2019, a dataset was constructed including those who lived in the Netherlands on 1 January and were at least 63 years old during that year.

For each year, information about sex, age and survival of all included persons was obtained. For the years 2020–2021 the population dataset was merged with a dataset containing information about all notified COVID-19 test results and a third dataset containing information about all vaccinations known by the National Institute for Public Health and the Environment (RIVM) for which written informed consent was given by the vaccinee to share these data (Supplemental Methods, Supplemental Table 1). During the study period COVID-19 was a notifiable disease, meaning that all cases had to be reported to the Municipal Health Services (GGD). However, large-scale testing facilities only became available in June 2020 and ended by the end of 2021, limiting our study period to the first two years of the pandemic. From April 2021 onwards, self-tests were available, which allowed for individual testing of which results were not reported or registered. However, until in 2022 it was highly recommended to confirm a positive self-test with a test at a GGD facility. The vaccination campaign started in January 2021 and was rolled out from old to young, with priority for certain high-risk groups and health care workers. By May 2021, all individuals of the age groups in our analysis had been invited for vaccination [8]. An estimated 7% of all vaccinated persons in the Netherlands did not give informed consent for sharing their vaccination data, although this percentage is lower for older persons [7, 9]. Because of this, about 8–25% of those considered as the unvaccinated group in our dataset were actually vaccinated (Supplemental Analysis 1). Furthermore, a few nursing homes did not share their vaccination data with the RIVM; their residents may therefore also be misclassified as unvaccinated in our dataset [9].

### Definitions

The starting day of a new COVID-19 infection period was defined as the day a positive COVID-19 test was obtained after a period of at least 90 days without a positive COVID-19 test. As the risk of COVID-19-related death decreases over time since infection [3], we made a distinction between the acute infection period, defined as the first 28 days of an infection period, and beyond [10]. Persons were considered to have a recent infection during the acute infection period and a non-recent infection after this period (until the start of a new infection, vaccination or death). The time of vaccination was defined as the day of the first SARS-CoV-2 vaccination. See Supplemental Analysis 1 for an estimation of how many vaccinations have been missed because of no consent.

### Statistical analyses

All analyses were performed for the total cohort and stratified per subset based on sex (men and women) and age groups based on age in years on 1 January 2020: 63–64, 65–69, 70–79, 80–89 and 90+. All models have calendar time as timescale (from 1 January or 1 June 2020 until 31 December 2021), as COVID-19 infections and related deaths occurred in waves [9]. The primary endpoint is excess mortality during 2020–2021, which can be expressed as a cumulative probability (absolute scale) and as a proportion of the total observed mortality (relative scale).

#### Relative survival, background mortality and total excess mortality

The goal of the analyses was to split the observed all-cause mortality in 2020–2021 into two parts, background and excess mortality, without relying on information on the cause of death. This can be done with relative survival models, which split each person’s hazard of dying (i.e., the instantaneous risk of dying) into a background hazard of dying based on demographic variables (usually called ‘population hazard’) and an excess (often called ‘disease-specific’) hazard of dying. In this study, the background mortality hazard can be interpreted as the counterfactual mortality hazard, i.e., the mortality hazard that would have occurred without COVID-19. The hazards can be used to calculate time-dependent probabilities of background and excess death which add up to the probability of the observed mortality, analogous to the competing-risks framework where cumulative incidences add up to the total failure probability. Since the excess hazard is the difference between the observed and the background mortality hazards, it can become negative, expressing that the observed mortality is lower than expected. This implies that also the ‘probability’ of excess death can become negative in this case and should be interpreted as the difference between two (real) probabilities.

The background hazard depends on a rate table which is typically derived from publicly available population mortality rates containing deaths per year, sex and age, under the assumption that these variables are sufficient to correctly determine the background hazard. Relative survival has mainly been applied in the cancer setting: the patients’ survival is compared with that of the general population in the same period, with the assumption that the patients are similar to the general population except for their disease and treatment. In the current study, the ‘patient population’ consists of the whole selected population during 2020–2021 and the ‘general population’ is the reference cohort during 2015–2019. Since the timescale is calendar time and the background mortality slightly fluctuates throughout the year, month of the year was used as an additional variable in the rate table. For this, using the reference population we calculated the background hazard stratifying by sex, age and month of the year and constructed our own rate table (Supplemental Methods). With this rate table, the excess mortality hazard and probability in 2020, 2021 and 2020–2021 were estimated. When calculating confidence intervals for the probabilities, the background hazard was assumed fixed. The excess death probabilities were translated to absolute numbers of excess deaths (rounded to the nearest multiple of 10) by multiplying the probability by the number of persons at risk on 1 January 2020, assuming no effect of migration as only a small group migrated (Supplemental Fig. 1).

To evaluate the impact of the used background hazards on the excess mortality estimate, sensitivity analyses were performed with rate tables ignoring month of the year or based on publicly available life tables (Supplemental Analysis 2).

#### Multi-state model

**Fig. 1:**
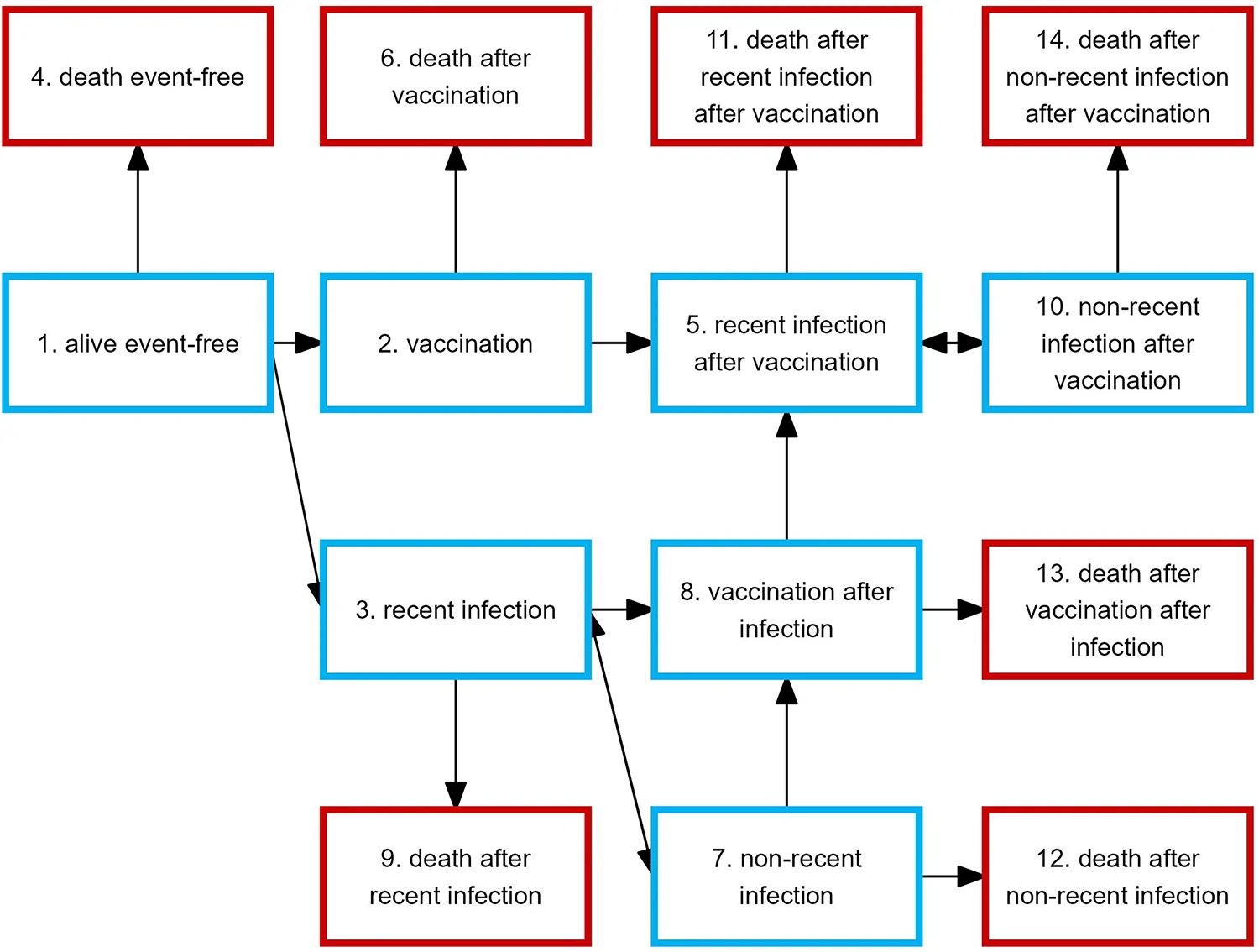
Structure of the multi-state model. Boxes show states, arrows transitions. All transitions to death states were split into background and excess by using relative survival. The number of persons in each non-death state over time is shown in Supplemental Fig. 2. The model only includes COVID-19 infections known by the GGD and COVID-19 vaccinations known by the RIVM for which informed consent was given to share these data

A multi-state model is a type of statistical model that describes processes where an individual transitions through a series of states over time. The aim of our multi-state model was twofold: to quantify the excess mortality taking place with and without documented infection or vaccination, and to investigate the impact of documented infection and vaccination on excess mortality. We constructed a non-parametric Markov time-inhomogeneous multi-state model, which considered the following events for its states: infection, vaccination and death. In a time-inhomogeneous Markov model, the hazard of making a certain transition only depends on the current state and the time since start. Thus, the hazard can vary over time, but the pathway is not considered. To incorporate part of the relevant history into the model, the infection state was split into four states, recent and non-recent infection with and without prior vaccination, and the vaccination state was split into vaccination with and without prior infection (Fig. 1). Because large-scale testing in the Netherlands only became available from 1 June 2020 onwards, the model starts on that date. Relative survival modelling was incorporated in this multi-state model in order to split all death states into background and excess death (e.g., background death after recent infection and excess death after recent infection), an approach recently developed by Manevski et al. [6]. By means of the Aalen-Johansen estimator the transition probabilities, representing the probabilities of being in a particular state at a point in time given starting state and time, were calculated based on the transition hazards. Confidence intervals for the probabilities of being in any of a set of states, such as all excess death states, were calculated based on the estimated variance-covariance matrix of all transition probabilities [11].

To investigate the impact of vaccination and infection on excess mortality, we compared the survival between persons who were in different states (e.g., vaccination or recent infection) at the same point in time. As time since infection had a strong impact on the risk of death, the Markov assumption that the transition hazard only depends on the current state and not on the time since entry in that state was violated for the transitions out of the infection-related states in our model. Therefore, we performed landmark analyses according to the landmark Aalen-Johansen method, which does not rely on the Markov assumption [12]. In the landmark Aalen-Johansen method, a landmark time is chosen (e.g., 1 September 2021) and only those who are still alive are included in the analysis. Per state in which persons were at that time, the multi-state model is fitted again until the end of follow-up, using the landmark time as the new starting time and the respective state as the new starting state, for only those who were present in that state at the landmark time and not allowing for delayed entry as in the Markov model. We adjusted this method by incorporating relative survival and ran the model from four landmark times (Supplemental Fig. 2).

As it takes some time to build up immunity after vaccination against SARS-CoV-2, we performed a sensitivity analysis with people changing their vaccination status 2 weeks after their actual vaccination date and repeated the landmark analyses for the total cohort.

#### Cumulative incidences

Cumulative incidences of first documented infection and vaccination were estimated in separate competing-risks models starting at 1 June 2020 with death as competing event. Only persons who were at risk at 1June 2020 were included.

### Software

All analyses were performed in R version 4.4.0 using the packages mstate [11] and relsurv [13]. All syntax is available on https://github.com/survival-lumc/COVID19_ExcessMortality.

## Results

### Evolution of excess mortality during 2020–2021

**Fig. 2:**
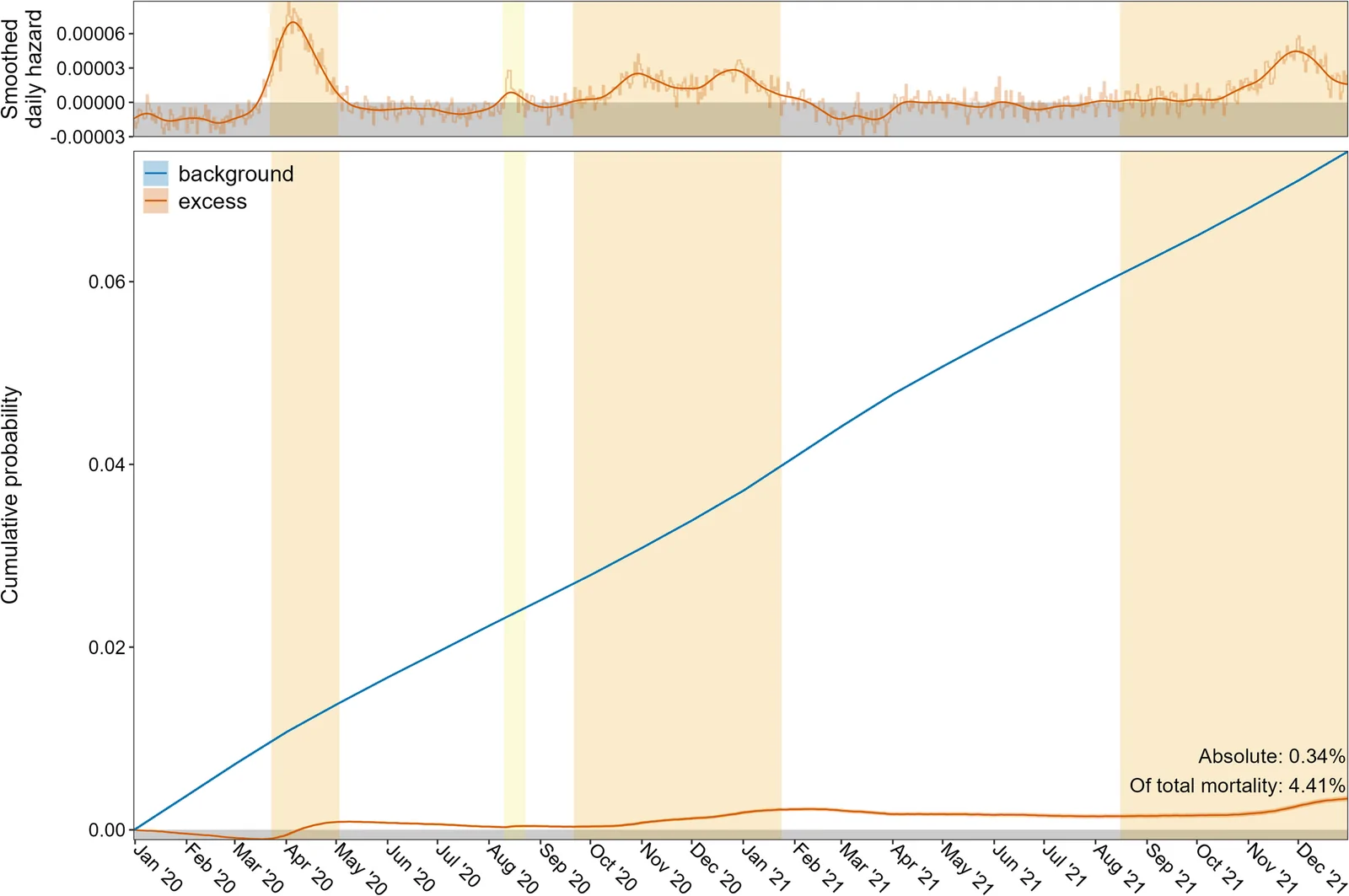
Daily hazard of excess mortality and cumulative probabilities of background and excess mortality during 2020–2021. The colored areas show the heat wave (yellow) and the three COVID-19 waves (light orange) in 2020–2021 as defined in the report of Statistics Netherlands and RIVM [9]. The upper panel shows the observed (light) and smoothed (dark) daily hazard of excess mortality. The lower panel shows the cumulative probabilities of background and excess mortality with 95%-CI. The label in the lower right corner gives the absolute and relative excess mortality probabilities until 31 December 2021

The study cohort consisted of 3,826,770 persons (Supplemental Table 2). To investigate the impact of the COVID-19 pandemic on mortality in the Netherlands in persons born before 1957, we fitted a relative survival model using 2015–2019 as reference years for the expected survival. As shown in Fig. 2, until the middle of March 2020 fewer persons died than expected, as indicated by a negative excess mortality hazard. During the first COVID-19 wave, from March until June 2020, more persons died than expected, resulting in a positive excess mortality hazard and an increasing excess mortality probability. After the first COVID-19 wave, the excess mortality hazard became negative again, which led to a slightly decreasing probability of excess mortality. The other two major increases in excess mortality coincided with the second and third COVID-19 waves. The absolute excess mortality for 2020–2021 was 0.34% (95%-CI 0.32–0.37), comprising 4.41% of the total mortality and corresponding to an estimated number of 13,120 excess deaths. The absolute excess mortalities for 2020 and 2021 separately were 0.19% (95%-CI 0.17–0.21, 4.83% of observed) in 2020 and 0.16% (95%-CI 0.14–0.18, 3.99% of observed) in 2021.

**Fig. 3:**
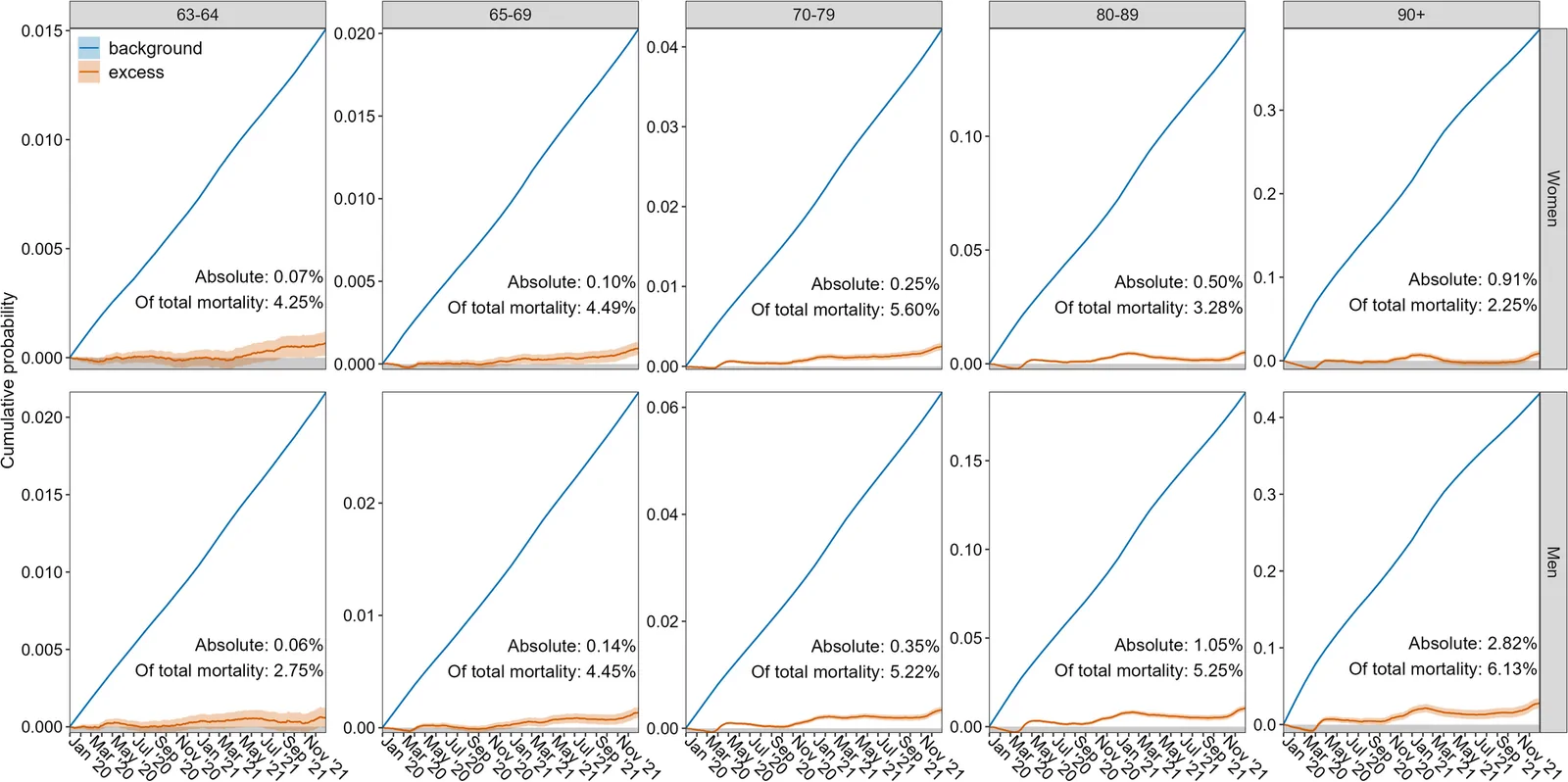
Cumulative probabilities of background and excess mortality with 95%-CI during 2020–2021 for the different subsets based on sex and age. The upper row shows the results for women, the lower row for men. The columns show the results for the different age groups (with age increasing from left to right). The labels in each panel give the absolute and relative excess mortality until 31 December 2021

The probabilities of excess death for the subsets based on sex and age are shown in Fig. 3. Absolute excess mortality was higher in men than women, except in the groups of 63–64 years old, and increased with age; it was lowest in men aged 63–64 (0.06%) and highest in men of 90 years and older (2.82%). There was a less clear association between age and excess mortality as proportion of the observed mortality, especially in women: for those of 63–64 years old, the excess mortality was 4.25% of the observed mortality, while the lowest relative excess mortality was observed in women of 90 years and older (2.25% of the observed mortality).

### Dynamics of infection by and vaccination against COVID-19 and excess death

For the evaluation of infection and vaccination, we considered the period from 1 June 2020 (start of large-scale testing, just after the first COVID-19 wave) up to and including 31 December 2021. At the end of this period, the cumulative incidences of documented infection and vaccination were 9.42% (95%-CI 9.40–9.45) and 89.13% (95%-CI 89.10–89.16), respectively (Supplemental Figs. 3, 4). Because the vaccination campaign in the Netherlands was mainly based on age, vaccination started earlier in older than younger persons (Supplemental Fig. 4).

**Fig. 4:**
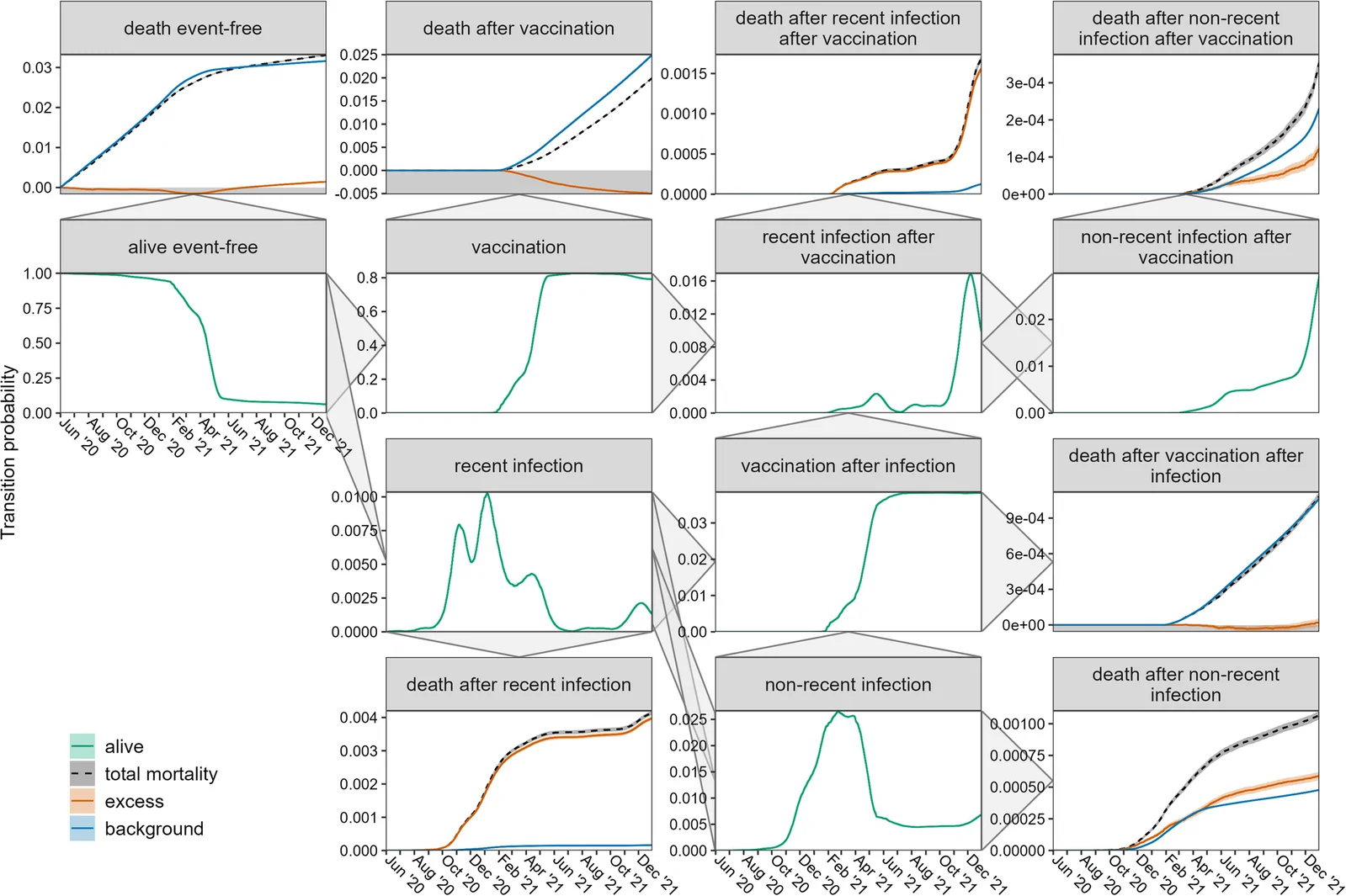
Transition probabilities with 95%-CI over time, based on the multi-state model in Fig. 1. The triangles on the panel sides show all possible transitions. All transitions to death are shown as the observed hazard and its two excess and background components. See Supplemental Table 3 for the exact values until 31 December 2021. Note that (non-)recent infection only includes documented infections

To investigate the dynamics of infection, vaccination and excess death, we integrated the relative survival model in the multi-state model shown in Fig. 1. From 1 June 2020 until the start of the second COVID-19 wave, the risks of becoming infected and dying of excess death were very low (Fig. 4). During the second wave, many persons became infected, after which the majority continued to the state non-recent infection. At the peak of this wave, in the first half of January 2021, the probability of currently having a recent documented infection was 1.03% (95%-CI 1.02–1.04). The risk of excess death during the recent documented infection phase was considerable: until the end of follow-up, the probability for the whole cohort was 0.40% (95%-CI 0.39–0.40), 96.07% of all deaths from this state (Supplemental Table 3). Even beyond the recent infection phase, unvaccinated persons died more often than expected: until the end of follow-up, the probability was 0.06% (95%-CI 0.06–0.06), 55.23% of all deaths after non-recent documented infection without prior vaccination. The vaccination campaign started in January 2021. At 1 June 2021, the probability of being in the vaccination state was 81.52% (95%-CI 81.48–81.56), while the probability of still being in the event-free state was 9.83% (95%-CI 9.80–9.86; note that this state also includes vaccinees who did not give informed consent for data sharing). By the end of follow-up, the probability of excess death after vaccination without subsequent documented infection was below zero (no infection before vaccination) to virtually zero (with infection before vaccination). During the peak of the third COVID-19 wave, in the first half of December 2021, the probability of currently having a recent documented infection with and without prior vaccination was 1.68% (95%-CI 1.67–1.69) and 0.21% (95%-CI 0.21–0.22), respectively. Vaccinated persons experienced excess death during the recent and non-recent documented infection phase as well: by the end of the follow-up the probabilities were 0.16% (95%-CI 0.15–0.16, 92.55% of observed) and 0.01% (95%-CI 0.01–0.01, 34.42% of observed), respectively. Absolute excess mortality without prior documented infection and vaccination was 0.14% (95%-CI 0.13–0.16, 4.36% of observed) at end of follow-up.

Supplemental Figs. 5–14 and Supplemental Table 3 show the outcomes for the different subsets. All subsets experienced excess mortality during recent and non-recent documented infection, including infection after vaccination, except women of 63–64 years old in the state non-recent infection after vaccination who had no excess mortality by the end of follow-up. Men aged 90 years or older with a recent documented infection without prior vaccination experienced most absolute excess mortality: 2.85% (95%-CI 2.67–3.03, 95.91% of the observed mortality during this phase). Of those in the state vaccination after documented infection, men of the age groups 80–90 and 90+ and women of the age groups 70–79, 80–89 and 90+ had excess mortality: 2.03% (men aged 80–89) to 20.49% (women aged 80–89) of their deaths were excess deaths. For all subsets, vaccinated persons without any documented infection had a negative excess death probability at the end of follow-up. However, in most subsets we observed a brief period of slightly positive excess mortality during the early months of 2021. To investigate this further, we repeated this analysis using subsets based on the birthyear cohorts used for the vaccination campaign [8], which indicated that this observation was merely the result of prioritizing vulnerable groups (Supplemental Analysis 3).

### Impact of infection by and vaccination against COVID-19 on excess mortality

**Fig. 5:**
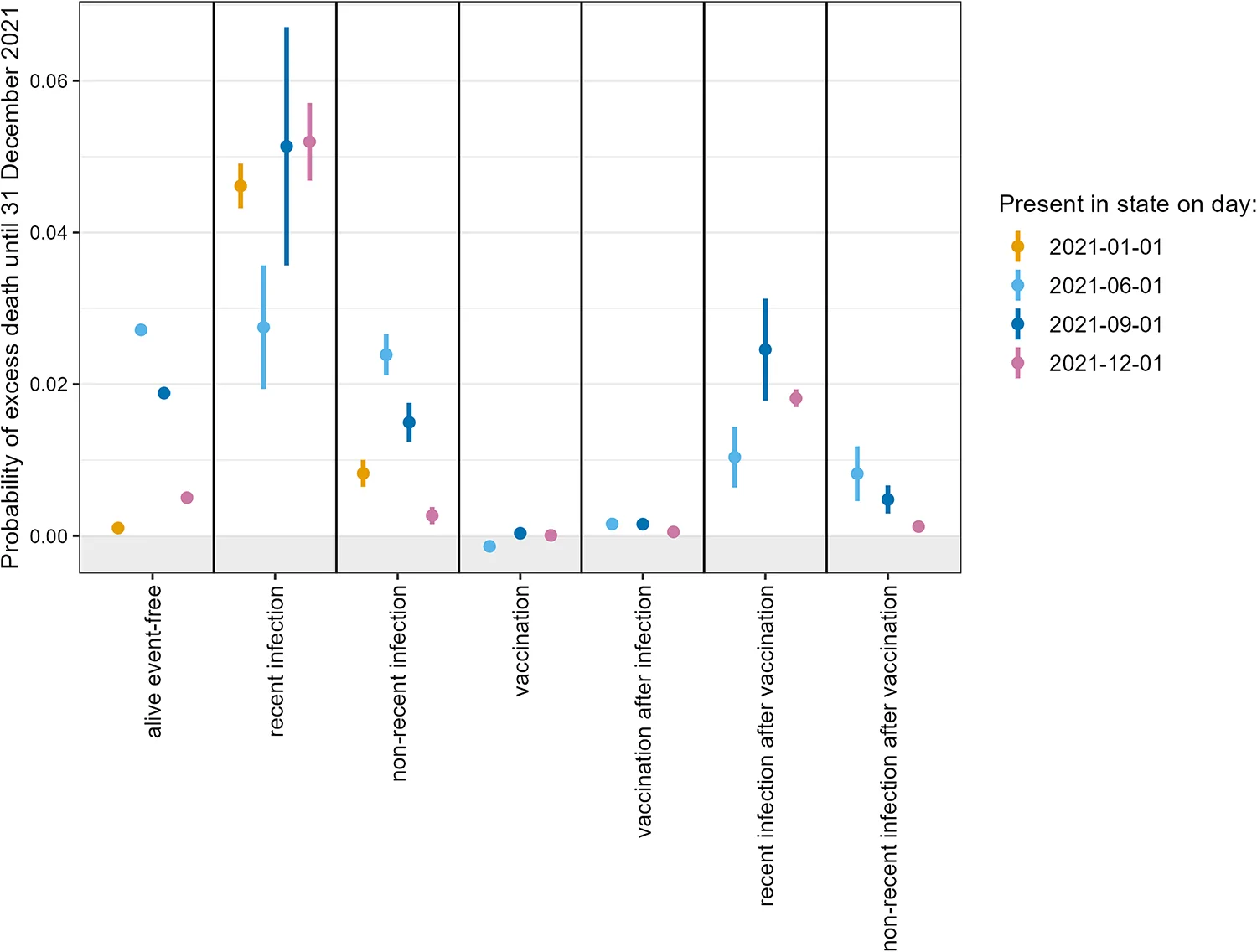
Excess mortality until 31 December 2021 with 95%-CI in the total cohort calculated from different states and landmark times. The excess mortality is the sum of all cumulative excess death probabilities. The x-axis shows the starting states, the colors show the landmark times. For instance, the first yellow dot from the left shows the excess mortality until 31 December 2021 for all persons who were in the state alive event-free on 1 January 2021. See Supplemental Table 4 for the exact values. The landmark analyses were only performed if at least 40 persons were at risk in the respective state and landmark time. Note that (non-)recent infection only includes documented infections

To investigate the impact of infection and vaccination on the risk of excess death until 31 December 2021, we performed landmark analyses from different states at different time points during the pandemic (Fig. 5, Supplemental Table 4, Supplemental Fig. 15). First, we analyzed the outcomes from 1 January 2021, which was during the second COVID-19 wave, before the vaccination campaign started. Persons with a recent documented infection at this time had the highest probability of excess death (4.61%, 95%-CI 4.32–4.91), followed by persons with a non-recent documented infection (0.82%, 95%-CI 0.65–1.00). Secondly, we analyzed the outcomes from 1 June 2021, which was in between the second and third COVID-19 wave and about a month after the last persons in our cohort had received their vaccination invitation. The probability of excess death was negative for persons in the vaccination state and highest for persons with a recent documented infection without prior vaccination (2.75%, 95%-CI 1.94–3.57), followed by those without any documented infection or vaccination (2.72%, 95%-CI 2.65–2.79) and those with a non-recent documented infection (2.39%, 95%-CI 2.12–2.66). Persons with a documented infection after vaccination had probabilities of 1.04% (recent infection; 95%-CI 0.64–1.44) and 0.82% (non-recent infection; 95%-CI 0.46–1.18). During the third COVID-19 wave, those with a recent documented infection without a prior vaccination at 1 September or 1 December 2021 had the highest probability of excess death (5.14%, 95%-CI 3.57–6.71, and 5.20%, 95%-CI 4.68–5.71, respectively), followed by those with a recent documented infection after vaccination (2.46%, 95%-CI 1.78–3.13, and 1.82%, 95%-CI 1.70–1.93, respectively). A sensitivity analysis with a 2-week delay in updating a person’s vaccination status to allow time for building up immunity after vaccination yielded very comparable results (Supplemental Fig. 16).

Also in the subset analyses by sex and age group, excess mortality was lowest from the vaccination states (with or without prior documented infection) and highest from recent documented infection without prior vaccination (Supplemental Fig. 17). The absolute differences were larger in older age groups and men (Supplemental Table 4).

To investigate the impact of vaccination on the risk of COVID-19 infection, we compared the probabilities of having a (non-)recent documented infection between those who were in the states alive event-free and vaccination at 1 June 2021. As shown in Supplemental Fig. 18, those who started in the vaccination state had lower probabilities of currently having a recent or non-recent documented infection from June to December 2021 than those who started in the state alive-event free. The impact of vaccination on the risk of documented infection was smaller than on the risk of excess death.

Taken together, these data show that excess mortality after a recent documented infection was considerable, and that beyond 28 days after infection, excess mortality was lower but still substantial. The data indicate a protective effect of being vaccinated: while excess mortality also occurred after infection after vaccination, this was much less than without prior vaccination.

## Discussion

### Interpretation of findings

This study quantifies excess mortality during the first two years of the COVID-19 pandemic in individuals aged over 63 in the Netherlands by means of advanced survival analysis methods. Excess mortality gives the net summary of the positive and negative effects of the pandemic on mortality (notably the impact of COVID-19 itself and (unintended) effects of the anti-COVID-19 measures on other diseases and events). By incorporating relative survival in a multi-state model, it provides insight in the impact of infection by and vaccination against SARS-CoV-2 on excess mortality. At the end of the study period, absolute excess mortality was 0.34%, comprising 4.41% of the observed mortality and corresponding to ca. 13,120 excess deaths. Excess mortality was observed in all sex and age groups, though certain periods and states showed lower-than-expected mortality.

We showed a positive association between documented infection and excess mortality which clearly increases with age on the absolute scale. Notably, the excess mortality relative to the background mortality showed a weaker association with age, especially in women whereby those of 90 years or older had the lowest relative excess mortality.

Even beyond 28 days after a positive COVID-19 test, the mortality was higher than expected. This is consistent with findings of Iwashyna et al., who reported excess mortality until 180 days after infection in a cohort of veterans (primarily during the first 90 days after infection), and Lam et al., who showed that adults still alive at 21, 30 or 90 days after infection had a higher risk of all-cause mortality compared to persons without COVID-19 [14, 15].

We showed a strong negative association between vaccination and excess mortality in all groups, as excess mortality after recent and non-recent documented infection was reduced in vaccinated persons. Vaccination was also associated with a lower risk of a documented COVID-19 infection, but as expected this association was weaker than with excess mortality. This is in line with other studies showing that SARS-CoV-2 vaccinations are most effective against severe outcomes [16–19]. Interestingly, we also observed excess mortality in the population without prior documented infection (or vaccination), which may be due to undiagnosed COVID-19 cases and indirect effects of the pandemic, or because those in the state alive event-free may on average have been unhealthier than those of the same sex and age who did receive a vaccination (the so-called ‘healthy vaccinee bias’) [4, 20]. The healthy vaccinee bias likely also leads to some overestimation of the differences between the excess mortality risks from the (non-)recent infection states with and without prior vaccination. This will be discussed further in the Limitations section below.

The observed negative association between SARS-CoV-2 vaccination and documented infection seems to be lower than in other studies [16, 17]. This is partly caused by our landmark-approach. This means that persons in the state alive event-free at the landmark time, i.e. the timepoint from which the landmark analysis started, could still receive a vaccination later, which likely influenced their infection risk. Moreover, in our study we only considered the first vaccination, while also the number of doses and time since the last vaccination are known to affect the protective effect of SARS-CoV-2 vaccinations [17].

### Methodological considerations

The quantification of excess mortality depends on the method, the model and which period is used as the reference. Especially in settings where the background mortality constitutes a large proportion of the total mortality, these choices can have a substantial effect. Inclusion of age in the background mortality is essential, because the risk of death increases with age as does the severity of a COVID-19 infection [3]. When choosing the reference period, it is important to take into account that the population mortality rate fluctuates over the years, also outside crisis periods, depending mainly on the severity of the yearly influenza season and the occurrence of extreme temperatures [21]. We decided to use 2015–2019 as the reference period in order to compare 2020–2021 to a period with averaged effects of the influenza season and temperature. Thus, in the definition of excess mortality used in the current paper, expected mortality includes mortality due to influenza and extreme temperatures. Because of the mild influenza season at the beginning of 2020 and the absent influenza season at the beginning of 2021, the excess mortality hazard was negative during these periods. In some other reported models, the impact of influenza on the background mortality was reduced by either ignoring periods with higher mortality caused by influenza [1], or by including the incidence of viral infections as a covariate [22]. This led to higher estimates of excess mortality, as the (unintended) protective effect of the anti-COVID-19 measures on other pathogens was taken less into account.

Multi-state models can capture complex sequences of events in a single comprehensive model, without suffering from the immortal time bias that occurs when baseline groups are naïvely determined by events occurring during follow-up. Combined with relative survival, multi-state models can be used to analyze the impact of intermediate events on excess mortality, without needing the exact causes of death and including any indirect effects of the pandemic on mortality. As the models have a continuous timescale, dynamics of events and outcomes can be followed in detail. Whereas this combined framework has previously been used to compare two populations in the same time period [6, 23, 24], we, for the first time, applied it to compare the same population in different time periods, e.g., under extreme circumstances and normal circumstances. The current study thus also serves as a proof of principle that the proposed model is suitable to analyze excess mortality under a crisis considering crisis-specific intermediate events. Moreover, we considered monthly trends in the background hazard, which is rarely taken into account in relative survival. Some technical aspects of our methodological approach are discussed in Supplemental Methods: Statistical considerations. Since the background risk of mortality is taken into account, absolute excess mortality can easily be compared between countries. All our syntax is publicly available to facilitate researchers who want to apply our models to similar data.

### Limitations

As Statistics Netherlands only has testing data available for 2020–2021, our study was limited to this period. Therefore, we could not investigate the dynamics of infection, vaccination and excess mortality during the later period of the pandemic.

Some concerns can be raised about confounding and bias in this study. During the study period COVID-19 was a so-called ‘notifiable disease’, meaning that all cases had to be reported to the GGD. However, before June 2020 there was a shortage of COVID-19 tests, leading to massive underreporting of the number of COVID-19 cases [7]. Since our model relies on COVID-19 test results, the multi-state analyses could only be started on 1 June 2020, when COVID-19 tests were widely available. In the model, all persons started on 1 June 2020 in the state alive event-free. This causes some bias in the hazard estimates to the states death event-free and recent infection, since persons who had been infected during the first COVID-19 wave might have experienced long-term effects of their COVID-19 infection and likely acquired immunity against COVID-19. Vos et al. reported after the first COVID-19 wave a seroprevalence of 4–7% in persons aged 35 years or older participating in a serosurvey cohort (median inclusion date 14 June 2020) [25]. Moreover, symptomatic persons with COVID-19 who did not test or only did a self-test are not classified as having COVID-19 in our dataset. Vos et al. reported a seroprevalence of 26% in November 2021 (although closer to 20% for those aged 60 years or older) [26], while the cumulative incidence of infection in our cohort was only 9% at the end of 2021, showing that we missed quite some undocumented infections. Persons with an undocumented infection most likely had mild or asymptomatic COVID-19 disease [27, 28], suggesting that the observed risks of excess mortality after recent and non-recent infection in the landmark analyses would likely have been lower if these untested persons with COVID-19 had been documented (but translating to higher probabilities for the whole population). While part of the asymptomatic group was tested because of for instance close contact with a known COVID-19 case, another part will have been missed.

A study by Meulman et al. indicated underreporting of COVID-19 infections among lower income groups: they were less likely to have a documented COVID-19 infection but had higher risks of COVID-19-related hospitalization and mortality [29]. Similar patterns were seen for people with lower education levels [29]. In our model, this might explain some of the excess mortality from the state alive event-free.

Vaccination was voluntary, and vaccine uptake was lower in groups with lower socio-economic status, non-western migration background, low trust in the government or strong religious beliefs [30]. These factors and vaccination hesitancy itself have also been associated with lower adherence to other preventive measures and/or higher infection risk [25, 31]. Also, people may have become less strict in social distancing after vaccination [32]. Aside from the choice to be vaccinated, vaccinees had to give informed consent for sharing their vaccination data. Our ‘unvaccinated’ group contains a considerable number of persons who actually had been vaccinated but did not give consent for data sharing (8–25% in the different subgroups, see Supplemental Analysis 1), and some nursing home residents whose vaccination has been incorrectly registered. The latter are likely on average more vulnerable than the other vaccinated persons, but as it only occurred in a few nursing homes this probably has no large impact on our estimates. The first (i.e., missing informed consent) likely attenuates the observed differences between the vaccinated and ‘unvaccinated’ persons. Van Werkhoven et al. showed that the estimates of vaccine effectiveness against COVID-19 hospitalization and intensive care unit admission in the Dutch 70+ population would have been higher when the non-consent vaccinees had been correctly classified [19]. We speculate that this would also hold for excess mortality. Lastly, there is likely some healthy vaccinee bias: persons could only receive a vaccination if they did not have fever, and patients with an acute medical problem were less likely to receive their scheduled vaccination on time. This selection effect may have led to a fitter vaccinated population, with a lower risk of dying than the average population [4]. The double contrast of unvaccinated/vaccinated and infected/not infected in our model gives an indication of the relative contributions of the healthy vaccinee bias and the protective effect of vaccination on excess mortality. Our landmark analyses from the vaccination state indicate a healthy vaccinee bias: we observed negative excess mortality in the analysis starting at June 2021 which disappeared at later landmark times. From the state alive event-free the opposite occurred: relatively high excess mortality in the analysis starting at June 2021 which decreased over time. In contrast, the differences between the excess mortality risks for those with a recent documented infection with and without prior vaccination were clearly present at each landmark time and did not decrease over time. This strongly indicates a protective effect of vaccination.

## Conclusions

Our study provides detailed insights in the dynamics of infection, vaccination and death during the first two years of the COVID-19 pandemic among individuals aged over 63 in the Netherlands. Our model allows to zoom in on specific subgroups, periods or states of interest. The absolute probabilities indicate that infection increased the risk of excess mortality whereas vaccination decreased the risk. The absolute risk differences increased with age, which supports the decision to first vaccinate the oldest. However, all groups experienced excess mortality, mostly during the acute phase of a documented COVID-19 infection, and had a clear survival benefit after being vaccinated. These results emphasize the essential role of vaccinations in reducing the risk of excess mortality during a pandemic.

## Supplementary Information

Below is the link to the electronic supplementary material.

Supplementary Material 1

## Data Availability

Results are based on calculations by the authors using non-public microdata from Statistics Netherlands. Under certain conditions, these microdata are accessible for statistical and scientific research. For further information: microdata@cbs.nl. All syntax of this study is available on https://github.com/survival-lumc/COVID19_ExcessMortality.
