# Supplementary material for: Dynamics of infection, vaccination and excess mortality during the COVID-19 pandemic among older individuals—a nationwide analysis
Source: Eur J Epidemiol. 2026 Jun 8;41(7):869–80. doi: 10.1007/s10654-026-01414-1 (PMC13572326; doi:10.1007/s10654-026-01414-1)
Supplement: Supplementary file 1 [file 10654_2026_1414_MOESM1_ESM.docx]

**Dynamics of infection, vaccination and excess mortality during the COVID-19 pandemic among older individuals – a nationwide analysis**

*European Journal of Epidemiology*

Eva A.S. Koster^1^, Marije H. Sluiskes^1^, Hein Putter^1^, Frits R. Rosendaal^2^, Astrid van Hylckama Vlieg^2^, Mark G.J. de Boer^2,3^, Liesbeth C. de Wreede^1^

1. Department of Biomedical Data Sciences, Leiden University Medical Center, Leiden, the Netherlands
2. Department of Clinical Epidemiology, Leiden University Medical Center, Leiden, the Netherlands
3. Leiden University Center for Infectious Diseases, Leiden University Medical Center, Leiden, the Netherlands

Corresponding author: Eva Koster, e.a.s.koster@lumc.nl

*Supplementary Information*

**Table of Contents**

Supplemental Methods............................................................................................................................................ 1

Data preparation........................................................................................................................................ 1

Background mortality hazards.................................................................................................................. 1

Statistical considerations........................................................................................................................... 1

Supplemental Analyses........................................................................................................................................... 3

1. Estimation of the percentage of misclassified persons among the ‘unvaccinated’ persons.................. 3

2. Impact of different mortality background hazards................................................................................ 4

3. Outcomes of the multi-state model stratified by birthyear cohorts....................................................... 5

References............................................................................................................................................................... 6

Supplemental Tables............................................................................................................................................... 7

Supplemental Figures............................................................................................................................................ 12

# Supplemental Methods

## Data preparation

In almost all cases, infection dates were based on the reported date of the first positive COVID-19 test after a period of at least 90 days without a positive COVID-19 test. In a few cases however, these reported dates were obviously incorrect: occurring before 2020 or after death. In these cases, the date of the registration of the test result in the GGD’s HPZone system was used instead. Dates of infection or vaccination occurring on the day of or after death were set to one day before death to enable the multi-state model to process both events. Events seemingly taking place after death were likely caused by reporting delays. Infection dates occurring on the day of first vaccination were set to one day before.

## Background mortality hazards

To calculate the background mortality hazard per sex, age and month, the data of the reference population was structured in long format: each person had one row for each month and year that the person was included in the dataset (i.e., being at least 63 years old at the beginning of the month and living in the Netherlands at the start of the year). Because in the microdata of Statistics Netherlands all birthdays are set to the first of the month, no additional rows were needed for changing age.

In order to have large enough groups for the ages >90 years, some persons were cloned in the dataset: e.g., for age 91, all persons aged 89, 90, 92 and 93 were added again with an artificial age of 91, creating a 5-year age group centered around the age of interest. As there are less 93-year olds than 89-year olds, all persons with the new age 91 were reweighted based on their sex and original age. This procedure was also performed for the ages 92-105, where persons older than 105 years were included in the group with new age 105.

Then, the cumulative hazard from the start of the month until the end of the month was calculated, stratifying for sex, age and month. Thus, the monthly hazards were averaged over the years 2015-2019. The mean daily mortality hazard per stratum was calculated by dividing the cumulative hazard at the end of the month (or at day 28 for February) by the number of days in the respective month (or 28 for February). These hazards were structured as a rate table that could be used by the relative survival models (using the same daily hazards for 2020 and 2021). In these models, persons with a higher age than which are present in the rate table are assumed to have the background hazard of the oldest age that is present: e.g., a person aged 106 has the same daily background mortality hazard as a 105-year old.

## Statistical considerations

Some subtle technical points are relevant in understanding differences in results between our and others’ work. In our model background and excess mortality are treated as competing events, such that the sum of the background and excess mortality is the total observed mortality. Their absolute probabilities are based on the cumulative hazards of both competing events, in contrast to methods where the excess mortality is calculated by comparing the observed and reference mortality probabilities directly. See Pohar Perme et al. [1] for a more detailed explanation of relative survival.

The background mortality hazard depends on the current demographic composition of the population (regarding sex and age). This makes the role of a time-dependent reference population essential: if during a COVID-19 wave mostly older persons die, the background mortality hazard would decrease, as the remaining population would on average become younger. In the beginning of 2020, the contrary occurred: because fewer older persons died than expected due to the mild influenza season, the population was on average older than the pre-pandemic population and the background mortality hazard increased. As a result, only after a substantial number of deaths during the first COVID-19 wave the excess mortality hazard became positive. In other words, our model takes the ‘gain’ of a few months longer survival with respect to the pre-pandemic period correctly into account. This is not possible if the reference population is not continuously updated to match the current demographic composition of the study population.

One of the main assumptions of relative survival models is that the study and reference groups are similar aside from their ‘disease’. In our case we assume that the general health (apart from the part related to COVID-19) and hazards of dying due to other causes than pandemic-related are similar for the study and reference populations, i.e., that the life expectancy in the absence of COVID-19 did not change between 2015 and 2021. This likely explains largely why our excess mortality estimates are lower than those reported by Statistics Netherlands and RIVM, which considered increasing life expectancy (i.e., a lower background hazard) in their model [2].

In general, the study population is subjected to a stronger selection mechanism than the reference population, due to their disease or, in this setting, the crisis in which they live. This implies that later in follow-up the study group may become less similar to the reference population, because on average, fitter persons are more likely to survive [3,4]. This may lead to negative excess hazards of death, leading to decreasing probabilities of excess death, possibly even below zero. This phenomenon is more often observed when relative survival is combined with a multi-state model, since the persons’ fitness may influence which states they reach [3,4]. This likely also occurs in our model, where excess mortality from the vaccination state was negative, probably mainly caused by the healthy vaccinee effect.

# Supplemental Analyses

## 1. Estimation of the percentage of misclassified persons among the ‘unvaccinated’ persons

To get some insight in how many persons were misclassified in our analyses as ‘unvaccinated’, an explorative analysis was performed to compare our data with the vaccination coverage as reported by RIVM [5]. Their numbers (only given as rounded percentages) are based on an anonymized dataset of GGD GHOR Nederland (national umbrella organization of the GGDs and Regional Medical Assistance Organizations) containing all SARS-CoV-2 vaccinations administered by the GGDs regardless of informed consent for data sharing, and all SARS-CoV-2 vaccinations administered by other parties (e.g., general practitioners, hospitals) for which informed consent was given. Hence, they only miss the vaccinations without consent for sharing that were administered by the other parties, and vaccinations that have not correctly been registered. 85% of all vaccinations of the basic series were administered at a GGD facility [5].

For different birthyear cohorts, RIVM calculated the percentage of persons who received at least one vaccination during 2021 by dividing the number of these persons by the total number of persons of that birthyear cohort who were living in the Netherlands on 1 March 2021 [5]. We repeated their analyses on our dataset. Persons born in 1956 were excluded from this analysis, since the RIVM report only gives the percentage for the birthyear cohort ‘1956-1960’, of which the majority was not included in our study. The difference between our percentages and those reported by RIVM gives a reliable estimate of how many persons vaccinated by the GGD who did not give consent for data sharing are misclassified as being unvaccinated in our study. As shown in the table below, 89% of those born before 1931 were vaccinated according to RIVM compared to 88% according to our data. The absolute difference of 1% represents those who were vaccinated but missed in our dataset. This is 8% of those who were classified as ‘unvaccinated’ in our dataset (1/12). For the different birthyear cohorts, the percentage of misclassified among the ‘unvaccinated’ varies between 8% and 25%. The actual percentage is likely slightly higher as those who were vaccinated by another party and did not give consent or whose vaccination was not correctly registered are still missing.

| **Birthyear cohort** | **% vaccinated as reported by RIVM [5]**  **(A)** | **% vaccinated based on our data**  **(B)** | **% of vaccinated according to RIVM that is recorded in our data**  **(C=B/A (%))** | **% missed**  **(D=A-C)** | **% not vaccinated according to RIVM**  **(100-A)** | **% not vaccinated according to our data**  **(E=100-B)** | **% misclassified among the ‘not vaccinated’ in our data**  **(D/E (%))** |
| --- | --- | --- | --- | --- | --- | --- | --- |
| <1931 | 89 | 88 | 99 | 1 | 11 | 12 | 8 |
| 1931-1935 | 93 | 91 | 98 | 2 | 7 | 9 | 22 |
| 1936-1940 | 94 | 92 | 98 | 2 | 6 | 8 | 25 |
| 1941-1945 | 94 | 93 | 99 | 1 | 6 | 7 | 14 |
| 1946-1950 | 94 | 92 | 98 | 2 | 6 | 8 | 25 |
| 1951-1955 | 93 | 91 | 98 | 2 | 7 | 9 | 22 |

## 2. Impact of different mortality background hazards

To investigate the impact of the used background mortality hazards, the excess mortality analyses were repeated using three different rate tables in sensitivity analyses. The first was calculated in a similar way as described in the **Supplemental Methods** except that ‘month’ was ignored and persons at their birthday started a new row with their updated age: the background mortality hazards per sex and age are thus averaged over the whole year and do not take seasonality into account. The other two rate tables were built using the life tables from the Human Mortality Database (HMD, see [www.mortality.org](http://www.mortality.org)) to derive the daily mortality hazard for each sex and age: one rate table only used the hazards of 2019, the other used the mean hazards of 2015-2019.

As shown in the figure below, using background hazards averaged over the year (dark blue curve) instead of per month (pink) led to almost similar probabilities at the ends of 2020 and 2021 but a different shape of the curve during the years: positive excess mortality during the respiratory infection season, more negative excess mortality during the summer and different estimates of the cumulative excess mortality during the first and second COVID-19 wave. The rate table based on the HMD life tables of 2015-2019 (light blue) yielded slightly lower excess mortality: the data are based on the same population, but HMD uses a different method of calculating the daily mortality hazard for the highest ages. Considering only the life table for 2019 for the background mortality (yellow) yielded substantially higher excess mortality, likely mainly because of the relatively mild respiratory infection season during this year [6].

The considerable differences have several reasons: because the time scale is calendar time, seasonality of the background mortality becomes more important than in settings with a disease-specific timescale (such as time from diagnosis or start treatment), especially when the study population is old and the excess mortality occurs in waves. Moreover, the background mortality comprises the largest part of the total mortality: small changes in the estimates of the background mortality hazard have a relatively strong effect on the estimates of the (small) excess mortality.

## 3. Outcomes of the multi-state model stratified by birthyear cohorts

In the main analysis we observed a short period of (slight) excess mortality from the vaccination state. As an explanation, it has to be considered that during the campaign vulnerable groups were prioritized (e.g., residents of long-term care facilities). As these persons likely have a higher mortality risk in general, excess mortality after vaccination should be assessed considering the different starting dates of the vaccinations for the prioritized and non-prioritized groups. Since the age groups used in our study do not follow the age cut-offs used for the timing of the vaccination invitations, we repeated the multi-state analysis with birthyear cohorts based on those used during the vaccination campaign [7].

The figure below shows the cumulative probability of excess mortality with 95%-CI from the vaccination state for these different groups, where the red line marks the start of the vaccination of non-prioritized persons: from this moment non-prioritized people can enter the vaccination state, and eventually become the majority of those in this state. Only the period in which any group had positive excess mortality is shown. In all groups, the positive excess mortality started during the period when only prioritized persons were being vaccinated. Soon after the start of vaccinating non-prioritized persons, the excess mortality probabilities started to decline, meaning that from that moment people died less than expected. These dynamics indicate that the temporary positive excess mortality is most likely caused by the selection of vulnerable people and not by a direct effect of the vaccination.

# References

1.	Pohar Perme M, de Wreede LC, Manevski D. What is relative survival and what is its role in haematology? Best Pract Res Clin Haematol. 2023; https://doi.org/10.1016/j.beha.2023.101474

2.	CBS, RIVM. Sterfte en oversterfte in 2020 en 2021. 2022. https://www.cbs.nl/nl-nl/longread/rapportages/2022/sterfte-en-oversterfte-in-2020-en-2021. Accessed 25 February 2024.

3.	van Breeschoten J, van den Eertwegh AJM, Hilarius DL, et al. Population mortality in advanced melanoma patients with and without response and progression; data from the Dutch Melanoma Treatment Registry. Eur J Cancer. 2023; https://doi.org/10.1016/j.ejca.2023.01.006

4.	de Boer AZ, Bastiaannet E, Schetelig J, et al. Breast cancer mortality of older patients with and without recurrence analysed by novel multi-state models. Eur J Cancer. 2022; https://doi.org/10.1016/j.ejca.2022.07.029

5.	RIVM. Vaccinatiegraad COVID-19 vaccinatie Nederland, 2021. RIVM-rapport 2022-0055, 2022; https://doi.org/10.21945/RIVM-2022-0055

6.	van Asten L, Harmsen CN, Stoeldraijer L, et al. Excess Deaths during Influenza and Coronavirus Disease and Infection-Fatality Rate for Severe Acute Respiratory Syndrome Coronavirus 2, the Netherlands. Emerg Infect Dis. 2021; https://doi.org/10.3201/eid2702.202999

7.	Pluijmaekers AJM, de Melker HE. The National Immunisation Programme in the Netherlands. Surveillance and developments in 2020-2021. RIVM rapport 2021-0055, 2021; https://doi.org/10.21945/RIVM-2021-0055

# Supplemental Tables

| **Datasets** | **Variables** | **Original variables used for identification** | **Comment** |
| --- | --- | --- | --- |
| All datasets | personal identifier | RINPERSOON | Used to link different datasets |
| [GBASTANDBEVOLKINGTAB](https://www.cbs.nl/nl-nl/onze-diensten/maatwerk-en-microdata/microdata-zelf-onderzoek-doen/microdatabestanden/gbastandbevolkingtab-woongemeente-1-1-t-m-31-12-t-1) | postcode | GBAPOSTCODE4GAAF1JANUARI | Used to identify whether person was living in the Netherlands at 1 January of each year |
| [GBAPERSOONTAB](https://www.cbs.nl/nl-nl/onze-diensten/maatwerk-en-microdata/microdata-zelf-onderzoek-doen/microdatabestanden/gbapersoontab-persoonskenmerken-van-personen-in-de-brp) | age group (based on age in years at 1 January 2020) | GBAGEBOORTEDAG, GBAGEBOORTEMAAND and GBAGEBOORTEJAAR | Birthdays are always set to the first of the month in microdata of Statistics Netherlands |
|  | sex | GBAGESLACHT |  |
| [GBAOVERLIJDENTAB](https://www.cbs.nl/nl-nl/onze-diensten/maatwerk-en-microdata/microdata-zelf-onderzoek-doen/microdatabestanden/gbaoverlijdentab-datum-van-overlijden-van-personen-ingeschreven-in-het-gba) | date of death | GBADatumOverlijden |  |
| [CIMS](https://www.cbs.nl/nl-nl/onze-diensten/maatwerk-en-microdata/microdata-zelf-onderzoek-doen/microdatabestanden/cims-covid-vaccinatie-informatie-en-monitoringssysteem) | date of vaccination | Vaccinatiedatum | Only the first vaccination is considered; if at or after date of death, set to one day before death |
| [GGDCOVID19BM](https://www.cbs.nl/nl-nl/onze-diensten/maatwerk-en-microdata/microdata-zelf-onderzoek-doen/microdatabestanden/ggd-covid19-besmettingsmeldingen) | date of positive COVID-19 test | DatumCOVID19testafname | Use Tijdstiprapportage-COVID19besmetting when missing or obviously incorrect (occurring before 2020 or after death); if at or after date of death, set to one day before death; if at day of vaccination, set to one day before vaccination |
|  | COVID-19 test result | TypeuitslagCOVID19test | Used to identify positive tests |

**Supplemental Table 1.** Overview of the used microdata tables and the variables.

| **Sex** | **Age** | **Total included** | **At risk at 1 January 2020** | **At risk at 1 June 2020** | **Censored at end of follow-up** |
| --- | --- | --- | --- | --- | --- |
| Women | 63-64 | 207383 | 207068 | 206443 | 203821 |
|  | 65-69 | 504335 | 503646 | 501502 | 492940 |
|  | 70-79 | 821111 | 820308 | 812734 | 783806 |
|  | 80-89 | 411194 | 410961 | 397102 | 348421 |
|  | 90+ | 95009 | 94985 | 85057 | 56408 |
| Men | 63-64 | 204688 | 204186 | 203243 | 199675 |
|  | 65-69 | 494871 | 493967 | 490872 | 478221 |
|  | 70-79 | 762559 | 761597 | 751019 | 711285 |
|  | 80-89 | 287866 | 287642 | 274568 | 230419 |
|  | 90+ | 37754 | 37735 | 33050 | 20390 |

**Supplemental Table 2.** Numbers of persons included and at risk in each of the subsets at 1 January 2020, 1 June 2020 and end of follow-up. Age categories are based on age in years on 1 January 2020.

|  | **State** | **Alive** | **Observed mortality** | **Background mortality** | **Excess mortality** |
| --- | --- | --- | --- | --- | --- |
| **Total** | Event-free | 6.24 (6.21-6.26) | 3.31 (3.29-3.32) | 3.16 (3.16-3.16) | 0.14 (0.13-0.16) |
|  | Recent infection | 0.13 (0.13-0.13) | 0.41 (0.41-0.42) | 0.02 (0.02-0.02) | 0.40 (0.39-0.40) |
|  | Non-recent infection | 0.69 (0.68-0.70) | 0.11 (0.10-0.11) | 0.05 (0.05-0.05) | 0.06 (0.06-0.06) |
|  | Vaccination | 79.03 (78.99-79.07) | 2.00 (1.99-2.02) | 2.50 (2.50-2.50) | -0.50 (-0.52--0.49) |
|  | Vaccination after infection | 3.83 (3.81-3.85) | 0.11 (0.11-0.11) | 0.11 (0.11-0.11) | 0.00 (0.00-0.01) |
|  | Recent infection after vaccination | 0.97 (0.96-0.98) | 0.17 (0.17-0.17) | 0.01 (0.01-0.01) | 0.16 (0.15-0.16) |
|  | Non-recent infection after vaccination | 2.97 (2.95-2.99) | 0.04 (0.03-0.04) | 0.02 (0.02-0.02) | 0.01 (0.01-0.01) |
| **Women 63-64** | Event-free | 8.06 (7.95-8.18) | 0.78 (0.74-0.81) | 0.70 (0.70-0.70) | 0.07 (0.04-0.11) |
|  | Recent infection | 0.23 (0.21-0.25) | 0.05 (0.04-0.06) | 0.00 (0.00-0.00) | 0.05 (0.04-0.06) |
|  | Non-recent infection | 1.07 (1.02-1.11) | 0.03 (0.02-0.04) | 0.01 (0.01-0.01) | 0.01 (0.01-0.02) |
|  | Vaccination | 79.80 (79.63-79.97) | 0.39 (0.36-0.42) | 0.45 (0.45-0.46) | -0.06 (-0.09--0.04) |
|  | Vaccination after infection | 4.89 (4.80-4.99) | 0.02 (0.01-0.02) | 0.03 (0.03-0.03) | -0.01 (-0.02--0.01) |
|  | Recent infection after vaccination | 1.22 (1.17-1.27) | 0.01 (0.01-0.02) | 0.00 (0.00-0.00) | 0.01 (0.01-0.02) |
|  | Non-recent infection after vaccination | 3.45 (3.37-3.53) | 0.00 (0.00-0.01) | 0.00 (0.00-0.01) | 0.00 (0.00-0.00) |
| **Men 63-64** | Event-free | 7.25 (7.13-7.36) | 1.04 (1.00-1.09) | 0.99 (0.99-0.99) | 0.05 (0.01-0.10) |
|  | Recent infection | 0.17 (0.16-0.19) | 0.08 (0.07-0.10) | 0.01 (0.01-0.01) | 0.08 (0.07-0.09) |
|  | Non-recent infection | 0.97 (0.92-1.01) | 0.05 (0.04-0.06) | 0.02 (0.02-0.02) | 0.03 (0.02-0.04) |
|  | Vaccination | 79.39 (79.21-79.56) | 0.54 (0.51-0.57) | 0.66 (0.66-0.66) | -0.12 (-0.15--0.09) |
|  | Vaccination after infection | 5.45 (5.35-5.55) | 0.02 (0.01-0.03) | 0.04 (0.04-0.04) | -0.02 (-0.03--0.02) |
|  | Recent infection after vaccination | 1.37 (1.32-1.42) | 0.03 (0.02-0.03) | 0.00 (0.00-0.00) | 0.02 (0.01-0.03) |
|  | Non-recent infection after vaccination | 3.64 (3.56-3.72) | 0.01 (0.01-0.02) | 0.01 (0.01-0.01) | 0.00 (0.00-0.01) |
| **Women 65-69** | Event-free | 7.52 (7.45-7.59) | 1.03 (1.00-1.05) | 0.96 (0.96-0.96) | 0.06 (0.03-0.09) |
|  | Recent infection | 0.16 (0.15-0.17) | 0.08 (0.08-0.09) | 0.00 (0.00-0.00) | 0.08 (0.07-0.09) |
|  | Non-recent infection | 0.85 (0.83-0.88) | 0.03 (0.03-0.04) | 0.02 (0.02-0.02) | 0.01 (0.01-0.02) |
|  | Vaccination | 81.65 (81.54-81.76) | 0.51 (0.49-0.53) | 0.60 (0.59-0.60) | -0.08 (-0.10--0.06) |
|  | Vaccination after infection | 4.33 (4.27-4.38) | 0.03 (0.02-0.03) | 0.03 (0.03-0.03) | 0.00 (-0.01-0.00) |
|  | Recent infection after vaccination | 1.03 (1.00-1.06) | 0.02 (0.02-0.03) | 0.00 (0.00-0.00) | 0.02 (0.02-0.03) |
|  | Non-recent infection after vaccination | 2.75 (2.70-2.79) | 0.00 (0.00-0.01) | 0.00 (0.00-0.00) | 0.00 (0.00-0.00) |
| **Men 65-69** | Event-free | 6.73 (6.66-6.80) | 1.46 (1.43-1.50) | 1.42 (1.42-1.42) | 0.05 (0.01-0.08) |
|  | Recent infection | 0.15 (0.14-0.16) | 0.14 (0.13-0.15) | 0.01 (0.01-0.01) | 0.14 (0.13-0.15) |
|  | Non-recent infection | 0.74 (0.71-0.76) | 0.07 (0.06-0.08) | 0.02 (0.02-0.02) | 0.04 (0.04-0.05) |
|  | Vaccination | 81.11 (81.00-81.22) | 0.76 (0.73-0.78) | 0.89 (0.89-0.89) | -0.13 (-0.16--0.11) |
|  | Vaccination after infection | 4.63 (4.57-4.69) | 0.03 (0.02-0.03) | 0.05 (0.05-0.05) | -0.02 (-0.02--0.01) |
|  | Recent infection after vaccination | 1.13 (1.10-1.16) | 0.04 (0.04-0.05) | 0.00 (0.00-0.00) | 0.04 (0.03-0.04) |
|  | Non-recent infection after vaccination | 2.99 (2.94-3.04) | 0.01 (0.01-0.01) | 0.01 (0.01-0.01) | 0.00 (0.00-0.01) |
| **Women 70-79** | Event-free | 6.15 (6.09-6.20) | 1.98 (1.95-2.01) | 1.88 (1.88-1.89) | 0.09 (0.06-0.12) |
|  | Recent infection | 0.12 (0.11-0.12) | 0.22 (0.21-0.23) | 0.01 (0.01-0.01) | 0.21 (0.20-0.22) |
|  | Non-recent infection | 0.61 (0.60-0.63) | 0.06 (0.05-0.07) | 0.03 (0.03-0.03) | 0.03 (0.03-0.04) |
|  | Vaccination | 82.42 (82.33-82.50) | 1.18 (1.16-1.21) | 1.40 (1.40-1.40) | -0.22 (-0.24--0.19) |
|  | Vaccination after infection | 3.51 (3.47-3.55) | 0.06 (0.05-0.06) | 0.06 (0.06-0.06) | 0.00 (0.00-0.01) |
|  | Recent infection after vaccination | 0.85 (0.83-0.87) | 0.08 (0.07-0.08) | 0.01 (0.01-0.01) | 0.07 (0.06-0.08) |
|  | Non-recent infection after vaccination | 2.75 (2.72-2.79) | 0.01 (0.01-0.02) | 0.01 (0.01-0.01) | 0.00 (0.00-0.01) |
| **Men 70-79** | Event-free | 5.30 (5.25-5.35) | 2.88 (2.84-2.92) | 2.81 (2.81-2.81) | 0.07 (0.03-0.10) |
|  | Recent infection | 0.10 (0.10-0.11) | 0.40 (0.38-0.41) | 0.01 (0.01-0.01) | 0.39 (0.37-0.40) |
|  | Non-recent infection | 0.54 (0.53-0.56) | 0.11 (0.10-0.11) | 0.04 (0.04-0.04) | 0.07 (0.06-0.08) |
|  | Vaccination | 81.16 (81.07-81.25) | 1.69 (1.66-1.71) | 2.08 (2.08-2.09) | -0.40 (-0.43--0.37) |
|  | Vaccination after infection | 3.55 (3.51-3.59) | 0.07 (0.07-0.08) | 0.08 (0.08-0.08) | -0.01 (-0.02-0.00) |
|  | Recent infection after vaccination | 0.98 (0.96-1.00) | 0.15 (0.14-0.16) | 0.01 (0.01-0.01) | 0.14 (0.13-0.15) |
|  | Non-recent infection after vaccination | 3.04 (3.01-3.08) | 0.03 (0.03-0.03) | 0.02 (0.02-0.02) | 0.01 (0.01-0.01) |
| **Women 80-89** | Event-free | 5.80 (5.73-5.88) | 6.36 (6.28-6.43) | 5.99 (5.99-6.00) | 0.36 (0.28-0.44) |
|  | Recent infection | 0.11 (0.10-0.12) | 0.77 (0.75-0.80) | 0.03 (0.03-0.03) | 0.74 (0.71-0.77) |
|  | Non-recent infection | 0.66 (0.63-0.68) | 0.21 (0.20-0.23) | 0.09 (0.09-0.09) | 0.12 (0.11-0.13) |
|  | Vaccination | 74.28 (74.15-74.42) | 4.21 (4.15-4.28) | 5.49 (5.48-5.50) | -1.28 (-1.34--1.21) |
|  | Vaccination after infection | 3.11 (3.06-3.16) | 0.28 (0.27-0.30) | 0.23 (0.22-0.23) | 0.06 (0.04-0.07) |
|  | Recent infection after vaccination | 0.77 (0.75-0.80) | 0.34 (0.32-0.36) | 0.03 (0.03-0.03) | 0.32 (0.30-0.33) |
|  | Non-recent infection after vaccination | 2.99 (2.93-3.04) | 0.09 (0.08-0.10) | 0.05 (0.05-0.05) | 0.04 (0.03-0.05) |

*Table continues on next page.*

| **Men 80-89** | Event-free | 4.82 (4.74-4.90) | 8.22 (8.12-8.32) | 7.80 (7.79-7.81) | 0.42 (0.32-0.53) |
| --- | --- | --- | --- | --- | --- |
|  | Recent infection | 0.09 (0.08-0.10) | 1.32 (1.28-1.36) | 0.04 (0.04-0.04) | 1.28 (1.24-1.33) |
|  | Non-recent infection | 0.51 (0.49-0.54) | 0.21 (0.19-0.22) | 0.10 (0.09-0.10) | 0.11 (0.09-0.13) |
|  | Vaccination | 72.14 (71.97-72.31) | 5.42 (5.34-5.51) | 7.06 (7.04-7.07) | -1.63 (-1.72--1.54) |
|  | Vaccination after infection | 2.63 (2.57-2.69) | 0.24 (0.23-0.26) | 0.24 (0.23-0.24) | 0.00 (-0.01-0.02) |
|  | Recent infection after vaccination | 0.77 (0.74-0.81) | 0.60 (0.57-0.63) | 0.03 (0.03-0.03) | 0.57 (0.54-0.60) |
|  | Non-recent infection after vaccination | 2.94 (2.87-3.00) | 0.09 (0.08-0.10) | 0.06 (0.06-0.07) | 0.03 (0.01-0.04) |
| **Women 90+** | Event-free | 5.40 (5.24-5.55) | 18.42 (18.16-18.68) | 17.95 (17.90-18.01) | 0.47 (0.18-0.75) |
|  | Recent infection | 0.14 (0.12-0.17) | 1.98 (1.88-2.07) | 0.13 (0.13-0.14) | 1.84 (1.75-1.93) |
|  | Non-recent infection | 0.72 (0.66-0.78) | 0.59 (0.54-0.65) | 0.37 (0.35-0.38) | 0.23 (0.18-0.28) |
|  | Vaccination | 53.01 (52.67-53.34) | 10.79 (10.58-11.00) | 13.19 (13.12-13.26) | -2.39 (-2.62--2.17) |
|  | Vaccination after infection | 3.18 (3.06-3.30) | 0.79 (0.73-0.85) | 0.75 (0.73-0.78) | 0.04 (-0.02-0.10) |
|  | Recent infection after vaccination | 0.82 (0.76-0.88) | 0.88 (0.82-0.95) | 0.09 (0.09-0.09) | 0.80 (0.73-0.86) |
|  | Non-recent infection after vaccination | 3.06 (2.94-3.18) | 0.22 (0.19-0.26) | 0.16 (0.15-0.17) | 0.06 (0.03-0.09) |
| **Men 90+** | Event-free | 4.40 (4.18-4.63) | 20.93 (20.49-21.37) | 19.85 (19.76-19.95) | 1.08 (0.60-1.56) |
|  | Recent infection | 0.10 (0.06-0.13) | 2.97 (2.79-3.15) | 0.12 (0.11-0.13) | 2.85 (2.67-3.03) |
|  | Non-recent infection | 0.48 (0.40-0.55) | 0.49 (0.42-0.57) | 0.28 (0.26-0.30) | 0.21 (0.14-0.28) |
|  | Vaccination | 51.47 (50.93-52.01) | 11.72 (11.38-12.07) | 14.79 (14.66-14.91) | -3.06 (-3.44--2.69) |
|  | Vaccination after infection | 2.28 (2.12-2.44) | 0.63 (0.55-0.72) | 0.60 (0.56-0.64) | 0.04 (-0.05-0.12) |
|  | Recent infection after vaccination | 0.61 (0.52-0.69) | 1.33 (1.21-1.45) | 0.08 (0.08-0.09) | 1.25 (1.13-1.37) |
|  | Non-recent infection after vaccination | 2.34 (2.18-2.50) | 0.24 (0.19-0.29) | 0.14 (0.13-0.16) | 0.10 (0.05-0.15) |

**Supplemental Table 3**. Transition probabilities (in %) with 95% confidence intervals corresponding to the values of the curves of **Figure 4** and **Supplemental Figures 5-14** until 31 December 2021. For instance for the states without infection or vaccination (‘event-free’), the ‘Alive’ column shows the probability of being in the state alive event-free and the three mortality columns show the probabilities for death event-free (death directly from the event-free state): total observed mortality and the excess and background components. Within each row, observed mortality is the sum of background and excess mortality. For each (sub)group, the percentages in the columns ‘Alive’ and ‘Observed mortality’ add up to 100%. Note that (non-)recent infection only includes documented infections.

|  | **Start state/ date** | **Alive event-free** | **Recent infection** | **Non-recent infection** | **Vaccination** | **Vaccination after infection** | **Recent infection after vaccination** | **Non-recent infection after vaccination** |
| --- | --- | --- | --- | --- | --- | --- | --- | --- |
| **Total cohort** | 1 Jan | 0.10 (0.08-0.12) | 4.61 (4.32-4.91) | 0.82 (0.65-1.00) | NA | NA | NA | NA |
|  | 1 Jun | 2.72 (2.65-2.79) | 2.75 (1.94-3.57) | 2.39 (2.12-2.66) | -0.14 (-0.15--0.12) | 0.16 (0.08-0.24) | 1.04 (0.64-1.44) | 0.82 (0.46-1.18) |
|  | 1 Sep | 1.88 (1.82-1.95) | 5.14 (3.57-6.71) | 1.50 (1.24-1.75) | 0.03 (0.02-0.05) | 0.16 (0.10-0.22) | 2.46 (1.78-3.13) | 0.48 (0.29-0.67) |
|  | 1 Dec | 0.50 (0.47-0.54) | 5.20 (4.68-5.71) | 0.27 (0.15-0.38) | 0.01 (0.00-0.01) | 0.05 (0.02-0.08) | 1.82 (1.70-1.93) | 0.12 (0.06-0.19) |
| **Women 63-64** | 1 Jan | 0.08 (0.04-0.12) | 0.00 (-0.36-0.37) | -0.19 (-0.43-0.04) | NA | NA | NA | NA |
|  | 1 Jun | 0.64 (0.52-0.76) | 0.73 (-0.61-2.07) | -0.02 (-0.29-0.25) | -0.05 (-0.08--0.02) | -0.14 (-0.25--0.02) | 0.51 (-0.43-1.45) | -0.18 (-0.55-0.19) |
|  | 1 Sep | 0.41 (0.30-0.52) | 1.77 (-1.03-4.58) | 0.15 (-0.19-0.49) | -0.03 (-0.05--0.00) | -0.08 (-0.17-0.00) | 0.25 (-0.76-1.26) | 0.12 (-0.19-0.43) |
|  | 1 Dec | 0.11 (0.05-0.18) | 2.07 (0.92-3.23) | 0.05 (-0.12-0.21) | -0.01 (-0.02-0.01) | -0.00 (-0.05-0.05) | 0.06 (-0.06-0.18) | -0.04 (-0.10-0.02) |
| **Men 63-64** | 1 Jan | 0.03 (-0.02-0.08) | 0.48 (-0.02-0.99) | -0.41 (-0.67--0.16) | NA | NA | NA | NA |
|  | 1 Jun | 0.73 (0.59-0.87) | 0.47 (-0.82-1.77) | -0.07 (-0.39-0.25) | -0.09 (-0.12--0.05) | -0.36 (-0.47--0.25) | -0.24 (-0.83-0.34) | -0.26 (-0.70-0.18) |
|  | 1 Sep | 0.53 (0.40-0.67) | 0.86 (-1.58-3.30) | 0.08 (-0.30-0.45) | -0.00 (-0.03-0.03) | -0.24 (-0.31--0.16) | 1.39 (-0.34-3.11) | -0.20 (-0.41--0.00) |
|  | 1 Dec | 0.08 (0.01-0.14) | 2.21 (0.97-3.45) | -0.04 (-0.17-0.10) | 0.00 (-0.01-0.02) | -0.07 (-0.11--0.03) | 0.13 (-0.02-0.29) | 0.11 (-0.05-0.27) |
| **Women 65-69** | 1 Jan | 0.07 (0.04-0.10) | 0.68 (0.31-1.05) | -0.07 (-0.29-0.15) | NA | NA | NA | NA |
|  | 1 Jun | 0.99 (0.89-1.10) | 0.60 (-0.35-1.56) | 0.59 (0.23-0.95) | -0.06 (-0.09--0.04) | 0.00 (-0.11-0.11) | 0.17 (-0.37-0.71) | -0.02 (-0.70-0.66) |
|  | 1 Sep | 0.53 (0.44-0.62) | 0.86 (-0.81-2.53) | 0.19 (-0.10-0.47) | 0.01 (-0.01-0.02) | 0.01 (-0.07-0.09) | 0.79 (-0.21-1.79) | 0.08 (-0.21-0.36) |
|  | 1 Dec | 0.13 (0.08-0.17) | 1.26 (0.60-1.92) | 0.03 (-0.09-0.16) | 0.00 (-0.01-0.01) | 0.02 (-0.03-0.06) | 0.28 (0.14-0.42) | 0.03 (-0.06-0.13) |
| **Men 65-69** | 1 Jan | 0.10 (0.07-0.14) | 1.68 (1.18-2.18) | -0.10 (-0.36-0.16) | NA | NA | NA | NA |
|  | 1 Jun | 1.45 (1.32-1.59) | 2.24 (0.62-3.85) | 0.85 (0.41-1.30) | -0.11 (-0.13--0.08) | -0.33 (-0.43--0.22) | 0.48 (-0.21-1.16) | 1.32 (0.09-2.54) |
|  | 1 Sep | 0.95 (0.83-1.07) | 2.73 (-0.42-5.88) | 0.60 (0.16-1.05) | -0.01 (-0.03-0.01) | -0.17 (-0.24--0.09) | -0.06 (-0.72-0.61) | 0.06 (-0.26-0.37) |
|  | 1 Dec | 0.24 (0.17-0.30) | 3.12 (2.04-4.20) | 0.01 (-0.14-0.17) | 0.01 (-0.01-0.02) | -0.05 (-0.09--0.01) | 0.52 (0.35-0.70) | -0.05 (-0.13-0.02) |
| **Women 70-79** | 1 Jan | 0.12 (0.09-0.15) | 2.99 (2.47-3.51) | 0.76 (0.43-1.08) | NA | NA | NA | NA |
|  | 1 Jun | 2.22 (2.09-2.36) | 1.79 (-0.09-3.68) | 2.31 (1.72-2.89) | -0.09 (-0.12--0.07) | 0.07 (-0.07-0.21) | 0.41 (-0.30-1.13) | -0.06 (-0.52-0.40) |
|  | 1 Sep | 1.42 (1.30-1.54) | 4.57 (0.97-8.17) | 1.33 (0.83-1.84) | 0.00 (-0.02-0.02) | 0.04 (-0.06-0.15) | 1.27 (0.12-2.41) | 0.16 (-0.13-0.45) |
|  | 1 Dec | 0.35 (0.29-0.41) | 3.34 (2.40-4.28) | 0.20 (-0.01-0.41) | 0.00 (-0.01-0.01) | -0.01 (-0.06-0.04) | 0.83 (0.65-1.01) | 0.06 (-0.05-0.16) |
| **Men 70-79** | 1 Jan | 0.13 (0.09-0.17) | 4.30 (3.66-4.95) | 0.57 (0.19-0.96) | NA | NA | NA | NA |
|  | 1 Jun | 3.21 (3.03-3.39) | 3.38 (0.98-5.78) | 3.04 (2.29-3.79) | -0.18 (-0.21--0.15) | -0.18 (-0.34--0.02) | 1.44 (0.48-2.40) | 0.48 (-0.15-1.11) |
|  | 1 Sep | 2.13 (1.97-2.29) | 6.73 (2.23-11.23) | 1.19 (0.59-1.78) | 0.00 (-0.02-0.03) | 0.03 (-0.10-0.16) | 2.65 (1.13-4.17) | 0.44 (0.07-0.81) |
|  | 1 Dec | 0.59 (0.50-0.68) | 5.78 (4.45-7.11) | 0.29 (0.01-0.57) | -0.00 (-0.02-0.01) | 0.02 (-0.05-0.09) | 1.55 (1.31-1.79) | 0.08 (-0.04-0.21) |
| **Women 80-89** | 1 Jan | -0.06 (-0.15-0.03) | 10.27 (9.08-11.46) | 3.77 (2.90-4.64) | NA | NA | NA | NA |
|  | 1 Jun | 4.75 (4.44-5.05) | 8.21 (2.46-13.96) | 6.53 (5.21-7.85) | -0.38 (-0.45--0.30) | 1.79 (1.35-2.24) | 4.58 (1.07-8.08) | 2.90 (1.72-4.08) |
|  | 1 Sep | 3.28 (3.01-3.55) | 9.22 (2.13-16.31) | 4.23 (3.10-5.36) | -0.00 (-0.06-0.06) | 1.35 (0.99-1.70) | 3.99 (1.35-6.63) | 1.74 (0.93-2.54) |
|  | 1 Dec | 0.97 (0.81-1.12) | 9.02 (6.99-11.05) | 0.89 (0.33-1.44) | -0.02 (-0.05-0.01) | 0.52 (0.31-0.72) | 3.95 (3.42-4.47) | 0.39 (0.09-0.69) |
| **Men 80-89** | 1 Jan | 0.27 (0.14-0.40) | 13.52 (11.80-15.24) | 1.58 (0.46-2.70) | NA | NA | NA | NA |
|  | 1 Jun | 7.87 (7.41-8.34) | 12.81 (5.50-20.12) | 6.11 (4.22-8.00) | -0.28 (-0.38--0.18) | 0.50 (-0.09-1.08) | 4.24 (-0.20-8.69) | 0.33 (-1.00-1.67) |
|  | 1 Sep | 5.73 (5.30-6.16) | 21.00 (10.15-31.86) | 2.35 (0.89-3.82) | 0.19 (0.11-0.27) | 0.28 (-0.18-0.73) | 7.81 (3.83-11.79) | 0.67 (-0.29-1.63) |
|  | 1 Dec | 1.62 (1.37-1.88) | 14.55 (11.25-17.84) | 0.81 (0.01-1.61) | 0.05 (0.01-0.10) | 0.11 (-0.14-0.36) | 5.54 (4.81-6.28) | 0.26 (-0.11-0.64) |
| **Women 90+** | 1 Jan | 0.02 (-0.33-0.37) | 12.78 (9.80-15.76) | 4.88 (2.38-7.37) | NA | NA | NA | NA |
|  | 1 Jun | 8.19 (7.31-9.07) | NA | 8.20 (4.99-11.41) | 0.30 (-0.02-0.61) | 2.64 (1.22-4.05) | 13.51 (0.95-26.07) | 1.80 (-1.63-5.23) |
|  | 1 Sep | 6.43 (5.61-7.25) | NA | 6.15 (3.22-9.08) | 0.93 (0.67-1.19) | 2.63 (1.45-3.82) | 3.88 (-3.40-11.17) | 1.49 (-1.00-3.98) |
|  | 1 Dec | 1.97 (1.45-2.49) | 20.32 (14.67-25.96) | 0.42 (-0.98-1.82) | 0.28 (0.14-0.43) | 0.47 (-0.17-1.12) | 9.59 (7.97-11.22) | 0.51 (-0.51-1.53) |
| **Men 90+** | 1 Jan | 1.12 (0.51-1.73) | 22.13 (16.15-28.11) | 2.06 (-2.67-6.79) | NA | NA | NA | NA |
|  | 1 Jun | 12.59 (10.94-14.23) | NA | 12.43 (5.68-19.17) | 0.45 (-0.09-0.99) | 2.30 (-0.49-5.09) | NA | 5.69 (-1.24-12.61) |
|  | 1 Sep | 8.98 (7.39-10.57) | NA | 7.20 (1.28-13.11) | 1.17 (0.72-1.62) | 1.05 (-1.18-3.28) | NA | 4.74 (-0.34-9.83) |
|  | 1 Dec | 2.64 (1.62-3.67) | 29.02 (17.43-40.60) | 2.77 (-0.95-6.50) | 0.25 (-0.00-0.51) | 0.72 (-0.61-2.04) | 16.81 (13.30-20.31) | 2.43 (0.07-4.80) |

**Supplemental Table 4.** Transition probabilities (in %) with 95% confidence intervals of excess death until 31 December 2021 based on the state occupied at different landmark times in 2021 (see also **Figure** **5** and **Supplemental Figure 17**). Landmark analyses were only performed if at least 40 persons were at risk in the respective state and landmark time (otherwise NA). Note that (non-)recent infection only includes documented infections.

# Supplemental Figures

Note that (non-)recent infection in the figures only includes documented infections.

3,822,095 started
in dataset on
1 January 2020

4,675 immigrated

4,224 emigrated

3,525,386 still alive on 31 December 2021

3,755,590 present in dataset on 1 June 2020

**Supplemental Figure 1**. Flow diagram with number of persons included on 1 January 2020, censored on 31 December 2021 and of those leaving/entering because of migration. See StatLine for the demographic composition during 2015-2021: <https://opendata.cbs.nl/statline/#/CBS/nl/dataset/03759ned/table?dl=B212A>.

**Supplemental Figure 2**. Number of persons at risk in all non-death states over time. The dashed lines show the four landmark times: 1 January 2021 (during the second COVID-19 wave and just before the vaccination campaign started), 1 June 2021 (about one month after the last persons of our population of interest had received a vaccination invitation), 1 September 2021 (just after the start of the third COVID-19 wave) and 1 December 2021 (around the peak of the third COVID-19 wave). Sample sizes smaller than 10 are excluded from the plot.

**Supplemental Figure 3**. Cumulative incidence of having at least one positive COVID-19 test for the total cohort and the different subsets based on sex and age. Death is treated as a competing event.

**Supplemental Figure 4**. Cumulative incidence of receiving at least one registered vaccination against COVID-19 for the total cohort and the different subsets based on sex and age. Relevant starting times in the vaccination campaign by birthyear or occupation/risk group are shown below the panel. Note that death is treated as a competing event, leading to lower cumulative incidences of vaccination in the older age groups compared to the younger age groups.

**Supplemental Figure 5**. Transition probabilities with 95%-CI over time for women aged 63-64, based on the multi-state model in **Figure 1**. The triangles on the panel sides show all possible transitions. All transitions to death are shown as observed and its two components excess and background mortality. See **Supplemental Table 3** for the exact values until 31 December 2021.

**Supplemental Figure 6**. Transition probabilities with 95%-CI over time for men aged 63-64, based on the multi-state model in **Figure 1**. The triangles on the panel sides show all possible transitions. All transitions to death are shown as observed and its two components excess and background mortality. See **Supplemental Table 3** for the exact values until 31 December 2021.

**Supplemental Figure 7**. Transition probabilities with 95%-CI over time for women aged 65-69, based on the multi-state model in **Figure 1**. The triangles on the panel sides show all possible transitions. All transitions to death are shown as observed and its two components excess and background mortality. See **Supplemental Table 3** for the exact values until 31 December 2021.

**Supplemental Figure 8**. Transition probabilities with 95%-CI over time for men aged 65-69, based on the multi-state model in **Figure 1**. The triangles on the panel sides show all possible transitions. All transitions to death are shown as observed and its two components excess and background mortality. See **Supplemental Table 3** for the exact values until 31 December 2021.

**Supplemental Figure 9**. Transition probabilities with 95%-CI over time for women aged 70-79, based on the multi-state model in **Figure 1**. The triangles on the panel sides show all possible transitions. All transitions to death are shown as observed and its two components excess and background mortality. See **Supplemental Table 3** for the exact values until 31 December 2021.

**Supplemental Figure 10**. Transition probabilities with 95%-CI over time for men aged 70-79, based on the multi-state model in **Figure 1**. The triangles on the panel sides show all possible transitions. All transitions to death are shown as observed and its two components excess and background mortality. See **Supplemental Table 3** for the exact values until 31 December 2021.

**Supplemental Figure 11**. Transition probabilities with 95%-CI over time for women aged 80-89, based on the multi-state model in **Figure 1**. The triangles on the panel sides show all possible transitions. All transitions to death are shown as observed and its two components excess and background mortality. See **Supplemental Table 3** for the exact values until 31 December 2021.

**Supplemental Figure 12**. Transition probabilities with 95%-CI over time for men aged 80-89, based on the multi-state model in **Figure 1**. The triangles on the panel sides show all possible transitions. All transitions to death are shown as observed and its two components excess and background mortality. See **Supplemental Table 3** for the exact values until 31 December 2021.

**Supplemental Figure 13**. Transition probabilities with 95%-CI over time for women aged 90 and older, based on the multi-state model in **Figure 1**. The triangles on the panel sides show all possible transitions. All transitions to death are shown as observed and its two components excess and background mortality. See **Supplemental Table 3** for the exact values until 31 December 2021.

**Supplemental Figure 14**. Transition probabilities with 95%-CI over time for men aged 90 and older, based on the multi-state model in **Figure 1**. The triangles on the panel sides show all possible transitions. All transitions to death are shown as observed and its two components excess and background mortality. See **Supplemental Table 3** for the exact values until 31 December 2021.

**Supplemental Figure 15**. Cumulative probability with 95%-CI of excess mortality over time, calculated for the total cohort from different states and landmark times. The excess mortality is the sum of all excess death probabilities. The landmark analyses were only performed if at least 40 persons were at risk in the respective state and landmark time.

**Supplemental Figure 16.** Excess mortality until 31 December 2021 with 95%-CI in the total cohort calculated from different states and landmark times, based on a sensitivity analysis with a 2-week delay in updating a person’s vaccination status after their first vaccination. The excess mortality is the sum of all cumulative excess death probabilities. The x-axis shows the starting states, the colors show the landmark times. The landmark analyses were only performed if at least 40 persons were at risk in the respective state and landmark time.

**Supplemental Figure 17**. Excess mortality until 31 December 2021 with 95%-CI in the different subsets calculated from different states and landmark times. The excess mortality probability is the sum of all cumulative excess death probabilities. The x-axis shows the starting states, the colors show the landmark times. Outcomes for women are shown in the upper row, outcomes for men in the lower row. Columns are per age group with age increasing from left to right. See Supplemental Table 4 for the exact values. Landmark analyses were only performed if at least 40 persons were at risk in the respective state and landmark time.

**Supplemental Figure 18**. Transition probabilities with 95%-CI of being in any of the recent infection states (i.e., recent infection and recent infection after vaccination) or non-recent infection states (i.e., non-recent infection, non-recent infection after vaccination and vaccination after infection) over time, based on the landmark analysis from 1 June 2021 from the states alive event-free and vaccination (total cohort).
